# Analysis of a Low-Cost EEG Monitoring System and Dry Electrodes toward Clinical Use in the Neonatal ICU

**DOI:** 10.3390/s19112637

**Published:** 2019-06-11

**Authors:** Mark O’Sullivan, Andriy Temko, Andrea Bocchino, Conor O’Mahony, Geraldine Boylan, Emanuel Popovici

**Affiliations:** 1 School of Engineering, University College Cork, Cork T12 K8AF, Ireland; atemko@ucc.ie (A.T.); E.Popovici@ucc.ie (E.P.); 2 Irish Centre for Fetal and Neonatal Translational Research, University College Cork, Cork T12 K8AF, Ireland; G.Boylan@ucc.ie; 3 Tyndall National Institute, University College Cork, Cork T12R5CP, Ireland; andrea.bocchino@tyndall.ie (A.B.); conor.omahony@tyndall.ie (C.O.); 4 Paediatrics and Child Health, University College Cork, Cork T12 DC4A, Ireland

**Keywords:** neonatal EEG, EEG electrode, dry electrode, MicroTIPs, microneedles, g.tec

## Abstract

Electroencephalography (EEG) is an important clinical tool for monitoring neurological health. However, the required equipment, expertise, and patient preparation inhibits its use outside of tertiary care. Non-experts struggle to obtain high-quality EEG due to its low amplitude and artefact susceptibility. Wet electrodes are currently used, which require abrasive/conductive gels to reduce skin-electrode impedance. Advances in dry electrodes, which do not require gels, have simplified this process. However, the assessment of dry electrodes on neonates is limited due to health and safety barriers. This study presents a simulation framework for assessing the quality of EEG systems using a neonatal EEG database, without the use of human participants. The framework is used to evaluate a low-cost EEG acquisition system and compare performance of wet and dry (Micro Transdermal Interface Platforms (MicroTIPs), g.tec-g.SAHARA) electrodes using accurately acquired impedance models. A separate experiment assessing the electrodes on adult participants was conducted to verify the simulation framework’s efficacy. Dry electrodes have higher impedance than wet electrodes, causing a reduction in signal quality. However, MicroTIPs perform comparably to wet electrodes at the frontal region and g.tec-g.SAHARA performs well at the occipital region. Using the simulation framework, a 25dB signal-to-noise ratio (SNR) was obtained for the low-cost EEG system. The tests on adults closely matched the simulated results.

## 1. Introduction

Electroencephalography (EEG) records the electrical impulses of the brain. It is an essential tool in the real-time assessment of brain health and the detection of abnormal neurological activity. The accurate measurement of EEG signals is of significant interest to both clinical and research domains. However, EEG monitoring entails the use of expensive equipment and clinical expertise that is only available in tertiary care facilities. Studies have shown the importance of EEG in correctly diagnosing abnormal neurological events, such as seizures in neonates. Without EEG monitoring, only 9% of seizures are correctly identifiable by clinical staff [1]. Interpreting EEG is a difficult task and must be conducted by experienced neurophysiologists [2]. However, neurophysiologists are seldom available to review the EEG in real-time. A study examining the delay between the onset of a seizure and the administration of the necessary medication showed that the entire process of preparing the patient, recording and interpreting EEG, and ordering medication takes on average over 2 h. This study was completed in a hospital with the appropriate resources, equipment, and personnel to monitor EEG [3]. Primary care and low-resource settings often have no means of monitoring neurological health. Abnormal neurological activity is often entirely missed or retrospectively diagnosed due to a lack of EEG resources. Both cases increase the risk of a poor outcome for the neonate [4].

**Figure 1:**
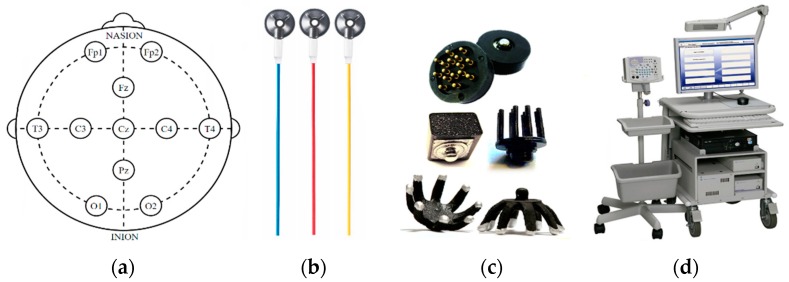
(**a**) Modified 10–20 system for neonates; (**b**) standard Ag/AgCl wet electrodes; (**c**) variety of dry electrodes; (**d**) EEG monitoring system.

The task of acquiring high quality EEG signals is non-trivial and requires the expertise of a neurophysiologist or EEG technician. Additionally, it is time-consuming, taking up to an hour in some cases [5,6]. EEG signals are very small in amplitude (±100 µV) and are susceptible to many types of artefacts, such as physiological, electrical, and movement artefacts that can often saturate the EEG signals with noise [7]. Therefore, to record accurate EEG signals, a lengthy patient preparation procedure is currently required, which involves the abrasion of the top layer of the skin and the application of conductive gels to improve the electrical conductivity [5]. The EEG monitoring process begins by locating the sites for electrode placement based on the international 10–20 system [8]. For neonates, a reduced electrode montage is often followed, as in Figure 1a [9]. The patient’s skin at these sites is then abraded with gel. This removes the top layer of the skin, the stratum corneum (SC), which is the largest contributor to the skin-electrode (SE) interface impedance. The SC impedance is inversely proportional to frequency, ranging from 200 kΩ–200 Ω over a frequency range of 1 Hz–1 MHz [10]. The most commonly used electrodes are flat or cup shaped metal (Ag/AgCl) electrodes, as in Figure 1b [9]. They must be coated with conductive gel to improve the electrical conductivity across the SE interface. Using this process, the SE impedance can be significantly reduced and a stable electrical connection between the electrode and skin can be established to capture the low voltage potentials. The standards of the International Federation of Clinical Neurophysiology (IFCN) state that SE impedances should remain below 5 kΩ throughout EEG recording [11]. Impedances below 5 kΩ are achievable using this procedure. The opposite end of the electrodes is connected to the amplifier and analog-to-digital converter (ADC) circuitry. The digitized EEG data is then processed by a computer and displayed on a monitor for interpretation by a neurophysiologist. Due to the sensitivity of EEG voltages, highly accurate electronics are required to ensure accurate EEG signals. This partly explains why clinical EEG systems are expensive. The cost of the system, its maintenance, and the clinical personnel required to operate the system significantly reduces its pervasiveness outside of tertiary care [12].

Alternative patient preparation techniques and electrode designs for adults have been thoroughly researched in recent years to facilitate quicker EEG electrode application. EEG head-caps and headsets provide ready-placed electrode holders positioned, according to the 10–20 system. Such devices are widely used in applications where high-density electrode montages are required [13,14]. Screen-printed electrodes are an alternative technology that provide comparable quality to conventional electrodes, while providing quicker and easier application [15,16]. Having fixed locations for electrodes can be a disadvantage, as it relies on all patients having similar head sizes. Such designs are of limited use on neonatal patients due to the variability in head sizes [17]. 

Dry electrode technologies rely on leveraging the use of novel mechanical designs to achieve improved connection with the skin, without the use of abrasive creams or conductive gels. There is a large variety of designs, as seen in Figure 1c. These include foam electrodes [18], polymer electrodes [19], and spring-based probes [20]. Arrays of nano, micro, and milli-needle dry electrode designs have been developed to penetrate the SC, significantly lowering impedance [21,22]. These technologies drastically reduce the amount of patient preparation and time required to record EEG.

Several studies assessing the performance of dry electrodes have shown comparable results between wet and dry electrodes using multiple evaluation experiments on adults. Such tests include simultaneously recording wet and dry channels and comparing correlation and coherence [6,20], or performing tasks such as generating an EEG alpha rhythm [19] and other neurological tasks [22,23]. 

There has been significant development in improving the cost, size, and usability of EEG monitoring systems. Many systems are moving to battery-powered and wireless solutions, which are usable across a multitude of applications and settings [24,25]. These developments further support the envisioned goal of wearable EEG [26]. The advocacy to provide open-source platforms has significantly aided advances [27,28]. Many of these systems have been evaluated with respect to gold standard machines outside of the clinical domain [29]. Though many novel EEG electrode and system technologies have been widely used in the brain computer interface and related research domains [30,31], their use in the clinical domain has been limited due to concerns regarding their accuracy and a lack of previous clinical use [32,33]. Clinically validating such systems requires using uncertified electronics on patients, which is often restricted by patient safety and ethical barriers [34]. In particular for newborns, there has been minimal development and evaluation of dry electrodes [6].

In this paper, a framework for assessing the quality of neonatal EEG acquisition systems is presented that bypasses the need for human volunteers by using an arbitrary waveform generator (AWG) outputting signals from a database of clinically obtained neonatal EEG. Previous studies using sinusoid signals to test EEG systems have shown that the performance varies depending on the frequency of the input signal [35]. Therefore, testing the system on actual EEG data provides a more accurate measure of performance [36,37]. The EEG simulation framework is used to compute the accuracy of an open-source, wireless EEG acquisition board and compare the performance of various dry electrodes to the gold standard wet electrodes using accurately obtained SE impedance models of each electrode. To verify the effectiveness of the simulation framework, the electrodes were tested on healthy adult volunteers and the results between both testing methods were compared.

The goals, experiments, and contributions of this paper are summarized below:

1. Investigation and modelling of skin-electrode impedance of dry and wet electrodes on adults.
2. Development of a neonatal EEG simulation test bench, using above impedance models.
3. In-vivo assessment of dry versus wet electrodes on adults, and a comparison of in-vivo results versus the proposed simulation framework.

This work forms part of the front-end design of a proposed portable, low-cost, and machine learning-assisted neonatal EEG acquisition and interpretation system [38,39]. The proposed system aims to increase the demographic of hospitals and clinicians that have access to EEG monitoring equipment and expertise. The portable hardware, used in collaboration with dry electrodes that are easy to apply, significantly simplifies the EEG acquisition process. In addition, the processing of EEG data using visual, audible, and machine learning algorithms on a smartphone or tablet facilitates intuitive EEG interpretation, leading to quicker diagnosis and treatment of possibly undiagnosed or misdiagnosed neurological abnormalities in neonates.

## 2. Materials and Methods

### 2.1. Skin-Electrode Interface

#### 2.1.1. Skin-Electrode Impedance Modeling

**Figure 2:**
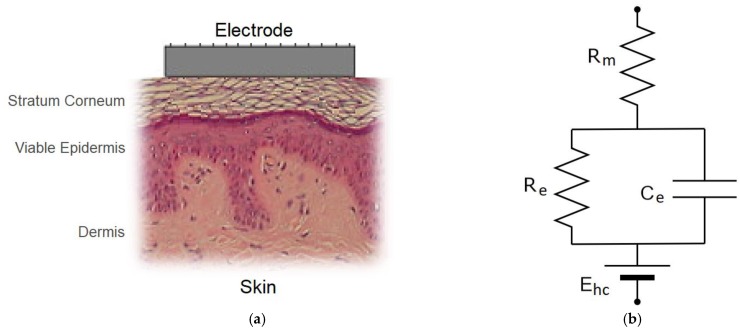
(**a**) Skin-electrode interface; (**b**) equivalent electrical model of skin-electrode interface

EEG signals must propagate from the brain, through the skull, skin, and hair before reaching the electrode. The amplitude of the scalp EEG potentials are generally in the range of ±100 µV. Therefore, throughout the duration of EEG recording, health care professionals constantly monitor the SE impedance to ensure that there is a stable and high-quality connection between the electrode and the skin, as in Figure 2a. The SE interface can be modeled using the simplified equivalent electrical circuit seen in Figure 2b. $E_{hc}$ is the half-cell potential of the electrode. This potential difference is caused by a difference in charge distribution between the electrode material and its surface electrolytes due to oxidization. $E_{hc}$ can be influential at low frequencies and under movement conditions. $E_{hc}$ can cause large DC voltage drifts, resulting in larger movement artefacts in the EEG signal [40]. $R_{m}$ is the resistance of the electrode material. $R_{e}$ and $C_{e}$ represent the impedance associated with the SE interface. At low frequencies, the circuit is dominated by the $R_{e}$ and $R_{m}$ series combination, as $C_{e}$ is large, acting as an open circuit. At higher frequencies, $C_{e}$ decreases, thus the impedance is dominated by $R_{m}$ [40]. At low frequencies, $R_{m}$ is negligible compared to $R_{e}$ [41]. Thus, for EEG (<50 Hz), the SE interface electrical model can be approximated as $R_{e}$. 

**Figure 3:**
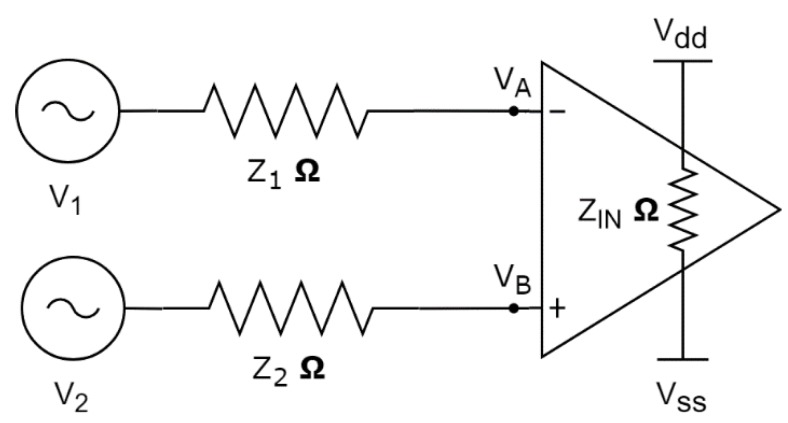
Skin, electrode, and amplifier interface electrical model.

Despite the IFCN recommending that $R_{e}$ remain below 5 kΩ throughout the EEG recording, due to the high input impedance of modern amplifiers (1 GΩ in the EEG acquisition board used in this study), the negative effects of high SE impedance are minimised [42]. The circuit in Figure 3 represents the electrical model of the skin, electrode, and amplifier. The EEG signals, $V_{1}$ and $V_{2}$, received by the scalp electrodes, are passed through the skin impedance models, $Z_{1}$ and $Z_{2}$ (simplified as $R_{e}$). The differential voltage received at the amplifier’s inputs ($V_{A}-V_{B}$) with an input impedance, $Z_{IN}$, can be computed using (1).

(1) $$V_{A}-V_{B}=\left(\frac{\left(V_{1}-V_{2}\right)}{2}\right)\left(2-\frac{\left(Z_{1}+Z_{2}\right)}{Z_{IN}}\right)+\left(\frac{\left(V_{1}+V_{2}\right)}{2}\right)\left(\frac{\left(Z_{1}-Z_{2}\right)}{Z_{IN}}\right)$$

Assuming that the SE impedance of the two electrodes, $Z_{1}$ and $Z_{2}$, are closely matched, the second term of the formula approaches 0. Taking the examples of typically low SE impedance (5 kΩ) and high SE impedance (100 kΩ), the respective signal losses can be calculated as follows:

(2) $$V_{A}-V_{B}=\left(\frac{\left(V_{1}-V_{2}\right)}{2}\right)\left(2-\frac{\left(5k\Omega +5k\Omega \right)}{1G\Omega }\right)=\left(V_{1}-V_{2}\right)\left(0.999995\right)=0.0005\%\;\mathrm{loss}$$

(3) $$V_{A}-V_{B}=\left(\frac{\left(V_{1}-V_{2}\right)}{2}\right)\left(2-\frac{\left(100k\Omega \;+\;100k\Omega \right)}{1G\Omega }\right)=\left(V_{1}-V_{2}\right)\left(0.9999\right)=0.01\%\;\mathrm{loss}$$

It is seen that the signal attenuation is 0.0005% in the presence of 5 kΩ SE impedance, and 0.01% in the presence of 100 kΩ SE impedance (as commonly achieved with dry electrodes). Thus, in theory, the attenuation of the EEG signal due to high SE impedances is minimal. The assumption that the SE impedances of all electrodes in the montage are closely matched (i.e., $Z_{1}=Z_{2}=Z_{N}$) is not always the case, especially with dry electrodes, as discussed in Section 4 of this paper.

#### 2.1.2. EEG Electrodes

**Figure 4:**
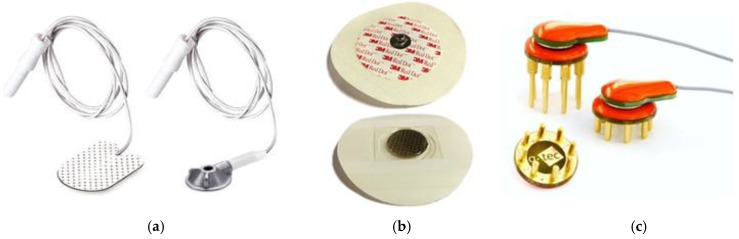
(**a**) Ambu Neuroline 700 and Cups; (**b**) MicroTIPs; (**c**) g.tec-g.SAHARA.

The electrodes used in this study are shown in Figure 4. The gold standard wet electrodes were represented by the use of abrasive gel (Nuprep Skin Prep Gel; Weaver and Company), conductive paste (Ten20; Weaver and Company), and Ambu Neuroline Cup or 700 electrodes. Using this process, SE impedances in the region of 5 kΩ are achievable. However, the process is time consuming and, in some cases, causes skin irritation [42]. This is particularly challenging for extremely preterm infants as their skin is very fragile.

Micro Transdermal Interface Platforms (MicroTIPs) are micro-moulded, polymeric microneedle structures that aim to penetrate the SC layer of the skin to make direct contact with the conductive layers of the epidermis beneath [43]. The TIPs are 500 µm in height, thus effectively penetrating the SC without contacting pain receptors or drawing blood. Unlike abrasive cream, MicroTIPs only pierce the SC at specific points, leaving the majority of the layer intact, thus reducing the risk of irritation or infection [42].

The g.tec-g.SAHARA electrodes are reusable, dry EEG electrodes for use over skin and hair. The electrodes consisted of eight 7 mm pins that are fabricated using a gold alloy. The electrodes are medically certified and can be used with g.tec’s active electrode leads, which contain preamplifier circuits that aim to improve signal quality [23]. In this study, the passive electrodes alone were used.

#### 2.1.3. Impedance Testing

As previously discussed, the SE impedance largely defines the quality of the EEG signals recorded. An SE impedance study was conducted on five healthy adult volunteers with varying skin and hair types. A frequency versus impedance sweep was repeated five times, at the frontal region (Fp1–Fp2) and occipital region (O1–O2) on each subject, for each electrode type, resulting in 300 impedance versus frequency sweeps in total. An Agilent E4980A LCR meter was used to setup the frequency sweep and measure the impedance response. The LCR meter injected an AC signal through one electrode and recorded the signal at a second electrode. The current of the signal was limited to 100 μA, in compliance with IEC60101 for patient leakage current [44]. 

The basic tolerance ($T_{B}$) of the meter used under the experimental conditions was 0.3%, as obtained from the datasheet [45]. The total tolerance of the system ($T_{T}$) can be calculated using (4). The test cable length ($L_{C}$) was 4 m and the frequency of interest ($F_{M}$) was 31 Hz, as many EEG systems use this frequency to perform impedance checks [46]. At low frequencies, this additional error was negligible. This method of impedance checking was far more accurate than the impedance measurement commonly used in low-cost EEG systems, which had a tolerance of roughly 20% depending on the accuracy of the current source [46].

(4) $$T_{T}=T_{B}+\left(0.015\%\times {\left(F_{M}/\left(1\times 10^{6}\right)\right)}^{2}{L_{C}}^{2}\right)$$

A 200-point frequency sweep over range of 20–1000 Hz was implemented. The resulting resistance ($R_{e}$) and reactance (X) values were recorded. Impedance (Z) was calculated using (5). The reactance in all cases was negative, denoting capacitive reactance. The capacitance ($C_{e}$) values were calculated using (6). The average Z, $R_{e}$, and $C_{e}$ frequency responses were calculated and graphed for each electrode at both frontal and occipital regions.

(5) $$Z=R_{e}+iX$$

(6) $$C_{e}=1/\left(2\pi f\left|X\right|\right)$$

### 2.2. System Framework

#### 2.2.1. Acquisition System

The amplitude of the signals captured by the electrodes are in the range of ±100 µV. Therefore, precise amplification and analog-to-digital conversion are required to maintain the integrity of the EEG signal. Differential amplifiers are often used as they amplify the difference between two input voltages, while suppressing any common voltages between them, such as electromagnetic interference (EMI), power-line noise (50 Hz), and DC bias. The common-mode rejection ratio (CMRR) of the amplifier is the amplifier’s effective suppression of the common voltages. The IFCN recommend a CMRR of 110 dB. Following amplification, the signal is digitized by an analog-to-digital converter (ADC). A minimum sampling rate of 200 samples per sec (Hz) is recommended, with a minimum resolution of 12 bits [11]. Custom system-on-chips (SoC) have been developed specifically for EEG recording to account for these challenges. The acquisition system used throughout this paper, the OpenBCI Cyton Bio-sensing board [28], utilizes the ADS-1299 chip [46] to acquire low-power (5 mW/channel) and low-noise (1 μVpp) EEG. The IFCN set instrumentation parameters that EEG acquisition systems should obey [11]. The OpenBCI board complies with all the standards (bar the minimum number of channels, which is irrelevant for the desired application), as shown in Table 1; however, the device is not currently approved for medical use under IEC60601-2-26 [47]. The input resistance ($R_{IN}$) and common-mode rejection ratio (CMRR) are discussed in depth in Section 4.

#### 2.2.2. EEG Simulation Framework

**Figure 5:**
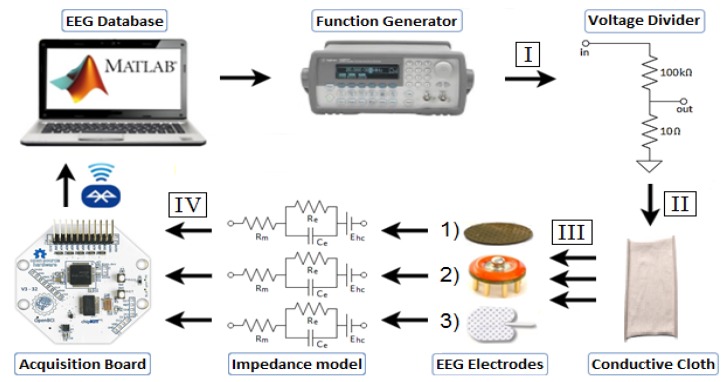
Simulation Framework.

The simulation setup is shown in Figure 5 [48]. The data used for simulation was obtained from a fully anonymized database of clinically acquired neonatal EEG at the Irish Centre for Fetal & Neonatal Translational Research (INFANT). The data was pre-processed in MATLAB to have a 250 Hz sampling rate, ±0.5 V amplitude, and a 0.5–100 Hz bandwidth. A 50 Hz notch filter was applied to remove power-line noise from the original recording. The data was split into 30-sec epochs. A total of 15 epochs were randomly selected, including healthy and non-healthy EEG. 

The digital data was passed to an Agilent 33220A arbitrary waveform generator (AWG), which converts the signal into the analogue domain. The amplitude of the output of AWG was scaled back to the original amplitude of the EEG signal (±100 μV) using a voltage divider circuit. The scaled signal was connected using crocodile clips to one end of the silver plated conductive cloth (Shieldex Techniktex P180; Statex) with less than 2 Ω/square [49]. The EEG electrodes were pressed on to the conductive cloth at the opposite end. The simplified passive electrical impedance models of the electrodes ($R_{e}$) for both frontal and occipital locations were attached to the output of the respective electrodes. Finally, the EEG acquired by each electrode was connected to an individual channel on the OpenBCI Cyton Bio-sensing board, which streamed the data to back to the laptop. The signals were processed in MATLAB for analysis and compared against the original data.

In order to individually account for the losses due to the simulation framework, the OpenBCI board, and the electrodes, the signals were recorded at multiple stages in the framework (stages I, II, III, and IV, in Figure 5), resulting in nine individual channels of EEG recordings. The output of the AWG (I) was recorded using a Tektronix MSO 3032 Oscilloscope. The remainder of the stages were recorded using the eight channels of the OpenBCI board. The output of the resistor divider (II) showed the accuracy of the recreation of µV EEG signals. The output of the conductive cloth (III) allowed for the calculation of losses due to the use of a 30 cm length strip of conductive cloth. At stage IV, the outputs of the three electrodes were connected to their respective impedance models, one model for frontal and one model for occipital. This resulted in six individual recordings, labelled as: Wet frontal (WF), wet occipital (WO), g.tec frontal (GF), g.tec occipital (GO), MicroTIPs frontal (MF), and MicroTIPs occipital (MO). 

The amplitudes of the resulting signals were normalized using z-score normallisation (7). The Pearson Correlation Coefficient (8) and the signal-to-noise ratio (SNR) (9) were computed, with respect to the original signal sent to the AWG for simulation. The power-line (50 Hz) noise was calculated by computing the Fast Fourier Transform (FFT) of the signal and obtaining the amplitude of the signal in the 50 Hz frequency bin.

(7) $$Z_{Y}=\left(\frac{Y-\mu_{y}}{\sigma_{y}}\right)\times \sigma_{X}+\mu_{X}$$

(8) $$r=\frac{1}{N-1}\sum_{i=1}^{N}\left(\frac{X_{i}-\mu_{x}}{\sigma_{x}}\right)\left(\frac{Y_{i}-\mu_{y}}{\sigma_{y}}\right)$$

(9) $$snr=20\log\left(\frac{X}{\left|X-Y\right|}\right)$$

where Y is the received signal, μ is the mean, σ is the standard deviation, and X is the original signals. The average and 95% confidence intervals (95% CI) values of correlation, SNR, and 50 Hz noise were calculated for each of the nine signals and averaged over the 15 epochs.

#### 2.2.3. In Vivo EEG

**Figure 6:**
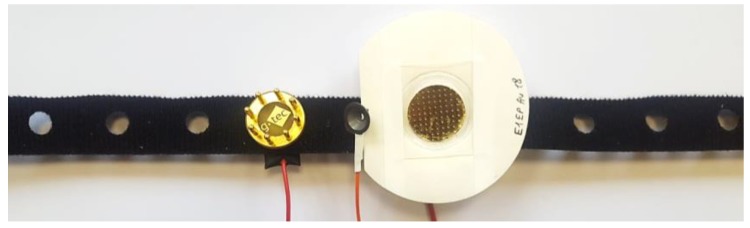
In vivo experiment electrode placement on OpenBCI electroencephalography (EEG) headband.

The most clinically objective method to compare EEG quality from dry electrodes is to compare it directly with wet electrodes on a human participant. With the approval of the Clinical Research Ethics Committee, three channels of EEG were simultaneously recorded on an adult volunteer using the OpenBCI board. The different electrodes (wet, g.tec, MicroTIP) were placed in close proximity to each other, with a 1 cm separation between the outer edges of the conducting surface of each electrode, as seen in Figure 6. The EEG was recorded from both the frontal (without hair) and occipital region (with hair). Ground and reference electrodes were placed on each ear lobe. The experiment was repeated five times on the same adult volunteer. The resulting signals were filtered (1–100 Hz) and notch filtered (50 Hz). The average correlation coefficients between the simultaneous wet and dry electrode signals were computed. 

It was expected that there would be minor variations in the EEG sources recorded by each electrode due to the 1 cm gap between them [50]. The electrodes were not moved relative to each other after each repetition, which may have also caused minor variations in correlation results.

## 3. Results

**Figure 7:**
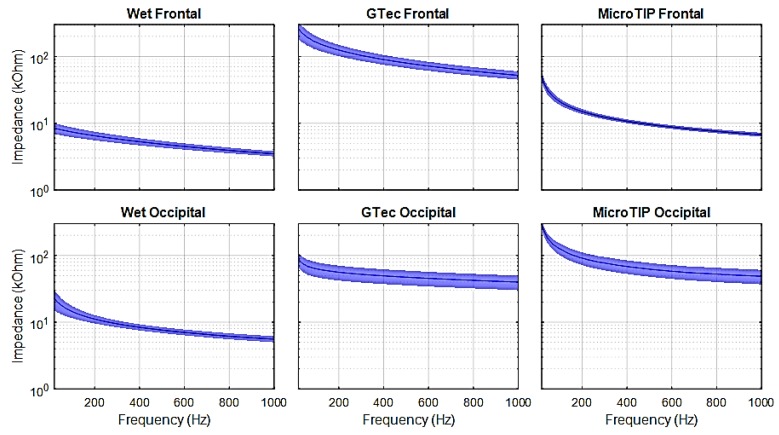
Skin-electrode impedance measured on healthy adult volunteers.

**Figure 8:**
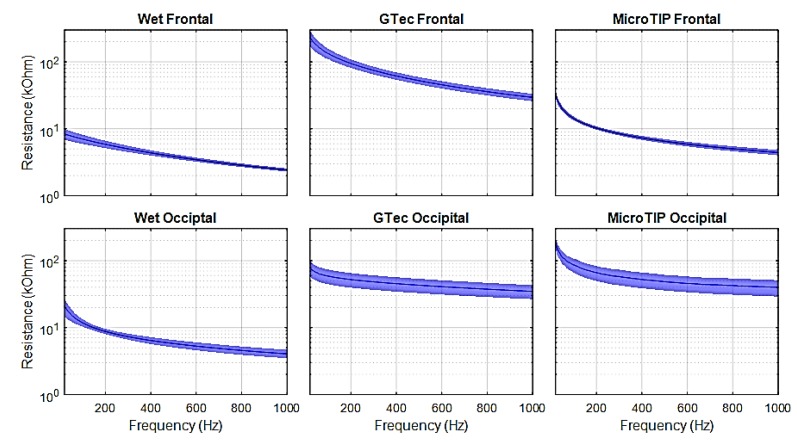
Skin-electrode resistance measured on healthy adult volunteers.

**Figure 9:**
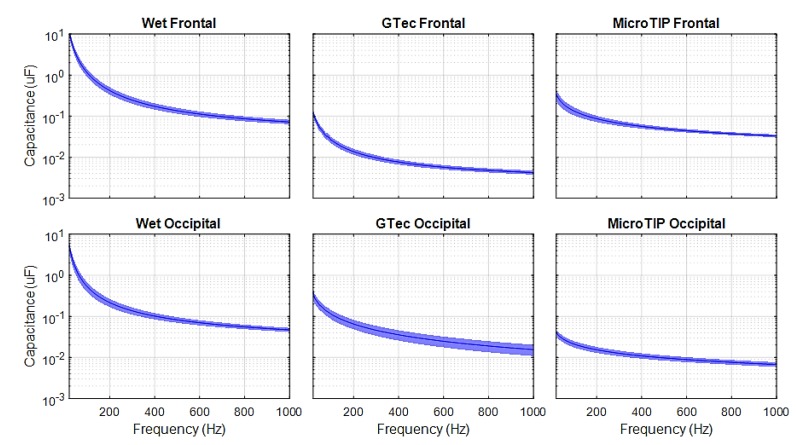
Skin-electrode capacitance measured on healthy adult volunteers.

Figure 7, Figure 8 and Figure 9 present the mean and 95% CI results of the SE impedance, resistance, and capacitance over a 200-point, 20-1000 Hz frequency sweep for each electrode at each location.

The specific impedance and resistance values for the 31 Hz test signal are presented in Table 2 for each electrode at both the frontal and occipital location. 

The results of the simulation framework are presented in Table 3. There are 9 channels of data in total, as described in Figure 5. The simulation experiment was repeated on 15 different 30-sec neonatal EEG segments. The mean and 95% CI over the 15 iterations was calculated for each channel. The correlation and SNR values were computed both on the unfiltered data and filtered data (50 Hz notch filter). The power noise (50 Hz) values were computed before filtering. Without additional filtering, the initial output signal from the generator, in the ±0.5 V range, achieved an SNR of 25 dB. The signal from the output of the resistor divider circuit, in the ±100 µV range, achieved an SNR of 23.4 dB and the signal on the cloth had an SNR of 22.7 dB. The losses in signal quality for the remaining channels were solely due to the electrodes and their respective SE impedance models. 

**Figure 10:**
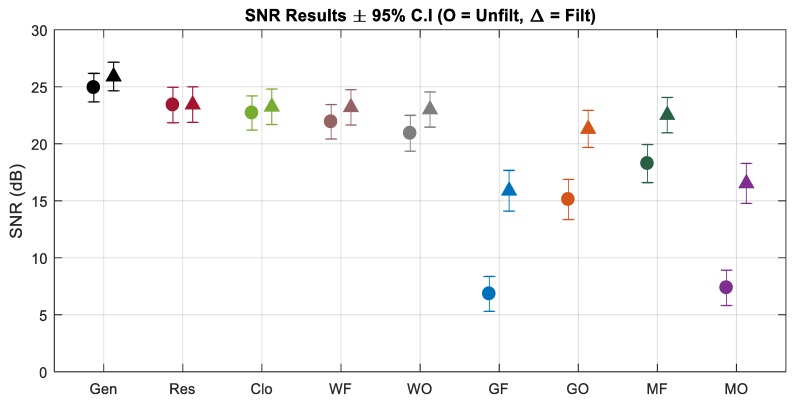
EEG simulation signal-to-noise ratio (SNR) results.

The average SNR results for each channel from the simulation framework are plotted in Figure 10. The circular and triangular markers represent the unfiltered and filtered results, respectively. In general, for electrodes with larger SE impedances, there is a greater loss in SNR and correlation. The power noise and signal quality losses are inversely related.

**Figure 11:**
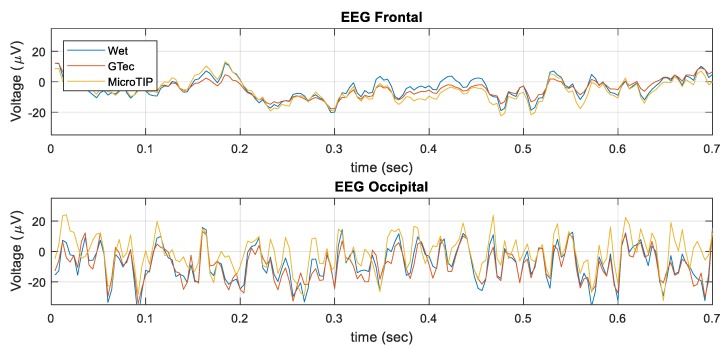
In vivo EEG segments.

The results of the in vivo experiments, comparing wet and dry electrode simultaneous EEG recordings, are presented in Table 4. The correlation values were calculated after band-pass (1–100 Hz) and notch (50 Hz) filtering. The MicroTIP electrode achieved a correlation of 0.92 at the frontal region. g.tec achieved a correlation of 0.86 at the occipital region. Figure 11 shows a section of EEG recorded by the electrodes at both the frontal and occipital region after filtering. On visual inspection, the EEG traces at the frontal region were closely matched for all three electrodes. At the occipital region, there were remnants of the 50 Hz artifact on the MicroTIPs and g.tec electrodes in particular, even after filtering.

## 4. Discussion

### 4.1. Skin-Electrode Impedance

Impedance versus frequency sweeps were completed on five adult volunteers, using three different electrodes at two locations on the head, and repeated 10 times in each case, resulting in 300 trials with lower than ±0.3% tolerance. The impedance values were given for a 31 Hz input signal (in-band) [51]. As expected, the wet electrodes achieved the lowest impedance due to the skin preparation procedure using abrasive cream and improved conductivity due to conductive gel. The average impedance using a 31 Hz test signal for both frontal and occipital regions were below 10 kΩ. These results are consistent with previous literature [43]. The g.tec electrode performed better on the occipital region (on hair) than on the frontal region. This is likely due to the reduced effective surface area of the conductor at the frontal region, where only the 2 mm-wide pins made contact with the skin. At the occipital region, the conductive plate behind the pins increased the effective surface area of the conductor, as salt-containing conductive solutions, such as oil and sweat in the hair, can make contact with the plate [52]. Conversely, the MicroTIP electrodes achieved lower impedances on the frontal region, as the stratum corneum (SC) was effectively penetrated by the 500 µm tips, which drastically reduced the impedance. These values were comparable with the gold standard wet electrodes. At the occipital region, the MicroTIPs were less effective, as the 500 μm tips struggled to maneuver through the dense hair and make contact with the SC. These impedance values were consistent with the results previously obtained using the MicroTIP electrodes for EEG [53] and for use on the arm for ECG recording [43]. The contribution of capacitive reactance to the overall impedance was higher with MicroTIPs compared to both wet and g.tec electrodes.

### 4.2. EEG Simulation Framework

The neonatal EEG database was almost perfectly recreated by the AWG. The output of the AWG achieved a correlation of 99.8% and an SNR of 25 dB with the original EEG data, without any additional filtering. Scaling the signal back to microvolt range and propagating it across the conductive cloth introduced losses, as expected, reducing the SNRs to 23.4 dB and 22.7 dB, respectively. These values closely match existing literature on the ADS1299, which found that, for 10–100 µV input signals, an SNR of 12–35 dB should be obtainable [54]. The passive impedance models for each electrode were added to the corresponding electrodes. The wet electrodes performed best in accurately recording the simulated EEG data, achieving an SNR of 22 dB and 21 dB for frontal and occipital impedances, respectively. The higher impedances of g.tec electrodes resulted in lower SNR values of 7 dB at the frontal region and 15 dB at the occipital region. The MicroTIPs performed relatively well at the frontal region, achieving an SNR of 18 dB. However, the high impedance at the occipital region resulted in a 7 dB SNR. 

The loss of signal accuracy with higher impedances was largely due to impedance mismatch of the signals entering the inputs of the differential amplifier’s inputs. In Equation (1), it is assumed that the input impedances are matched, hence there was minimal loss in accuracy, theoretically. Removing this assumption results in greater signal loss as the CMRR of the amplifier was dependent on the unwanted signals (such as the DC bias and 50 Hz noise) having the same amplitude and phase on both inputs. Introducing the larger impedances of the dry electrodes increased the impedance mismatch at the inputs of the differential amplifier. Thus, CMRR was reduced and the recordings were more susceptible to noise and interference. This was confirmed by the power noise results, which showed that there was a higher 50 Hz artefact in the higher impedance recordings. After applying a 50 Hz notch filter, the correlation and SNR values were re-calculated. The results improved drastically. g.tec electrodes achieved SNR values of 16 dB and 21 dB for frontal and occipital, respectively. The MicroTIPs achieved 22.5 dB and 17.5 dB, respectively. Thus showing that the reduced accuracy of dry electrodes was largely due to an increased impedance mismatch, resulting in lowered CMRR. 

The obtained correlation and power noise values are comparable to previous studies on alternative dry electrodes, which presented correlation values of roughly 0.99 and power-line noise of 0.5 μV [19]. Previous work investigating MicroTIPs for ECG recording on the arm achieved SNR values of 30.3 dB [43]. Considering the fact that the ECG was significantly larger in amplitude (±0.4 V) compared to EEG (±100 µV), the figures achieved in this study for MicroTIPs closely match the existing literature.

There are certain limitations and assumptions made in the simulation framework. It is assumed that the artifacts introduced by the skin-electrode interface were largely due to resistive impedance. Although, in Figure 2b, *R_m_* is considered negligible compared to *R_e_*, previous studies have shown that the composition of the electrode material and its polarization can directly affect the signals susceptibility to motion artifacts. Non-polarizable materials result in lower skin-electrode impedance at low frequencies [40]. This increased impedance at low frequencies is clearly evident in this study, as seen in Figure 7. The impedance models used in the simulations are from adults, not neonates. There are differences between adult and neonatal skin impedance and skull density [14]. Adult impedances would be higher than that of neonates, so the results in this study can be considered as the worst case for neonates. The literature states that the standard practise for both populations is to maintain impedance below 5 kΩ [11,55]. In addition, the EEG database used in this study is scalp EEG. Intracranial EEG (iEEG) would be more ideal, as it would not contain artefacts such as sweat, movement, and the low-pass filter effect the skin and skull has on the EEG signals. However, conducting iEEG recordings on neonates is not common practice and iEEG databases are not readily available.

### 4.3. In-Vivo EEG

The tests on adult volunteers provided additional validation of both the electrode results and the accuracy of the proposed simulation framework. The electrodes were simultaneously recorded five times at the frontal region and five times at the occipital region. The recorded signals were band-pass filtered (1–100 Hz), notch filtered (50 Hz), and de-trended. 

The correlation between the wet electrode (gold standard) and each of the dry electrodes was subsequently calculated. g.tec electrodes achieved correlation values of 83% and 86% for the wet electrode for frontal and occipital region, respectively. The MicroTIP electrodes achieved values of 92% and 78%, respectively. The trend of the results closely matched that of the simulation results. g.tec performed well over hair, and MicroTIPs performed well on bare skin. As expected, the values were lower than that of the simulation, as the simulation could not account for movement artefacts or other ambient conditions. In addition, due to the distance between the electrodes and distance from the reference electrode, minor discrepancies may have occurred due to variations in EEG sources.

Analyzing the EEG signals visually, it is notable that the wet electrode signal was less susceptible to movement and low-frequency major artefacts than the dry electrodes. As previously discussed, diminished performance under movement conditions was largely due to polarization. It is visible in Figure 11 that the occipital EEG signals still contained 50 Hz interference, in the dry electrodes in particular, even after applying a Finite Impulse Response (FIR) notch filter.

## 5. Conclusions

This work forms part of the front-end design of a proposed portable, low-cost, and user-friendly neonatal EEG acquisition and interpretation system. The paper presents an accurate and low-cost platform for assessing the accuracy and quality of EEG recording equipment without the need for human participants. This negates the concerns related to obtaining health/safety and ethical approval, which is required for human testing. Thus, the development iterative revision and improvement of novel EEG systems becomes much quicker and easier. The intrinsic losses in the simulation framework itself due to the digital-to-analog conversion and down-scaling were minimal. The low-cost, portable EEG acquisition system used in this study achieved high accuracy, with respect to the original EEG signal from the database. The study evaluated the use of dry electrodes compared to that of wet electrodes. The use of dry EEG electrodes resulted in higher skin-electrode impedances, as expected. However, micro-machined structures, such as MicroTIPs, effectively reduced the impedance, particularly in regions without hair. The use of larger pins, such as in g.tec-g.SAHARA electrodes, reduced the impedance over the hair. Introducing these impedances into the simulation experiment had a drastic impact on the EEG signal due to an impedance mismatch and signal attenuation. The inclusion of impedance models into the simulation framework developed a more realistic scenario for testing EEG equipment. The results show that, with the use of additional filtering, large impedances do not corrupt the EEG signals enough to significantly affect the intelligibility of the EEG signal.

Providing a quickly applicable EEG recording system to medical staff to assess the brain immediately after birth and during suspected abnormal neurological activity will improve the care and outcomes for neonates. This paper assists the development and testing of portable and user-friendly EEG technologies for the neonatal population.

## Figures and Tables

**Table 1:** International Federation of Clinical Neurophysiology (IFCN) standards versus OpenBCI specifications.

	Sample Rate (Hz)	Resolution (bits)	$R_{IN}$ (MO)	CMRR (dB)	Cross-Talk (dB)
IFCN	200	12	100	110	40
OpenBCI	250	24	1000	120	110

**Table 2:** Skin-electrode (SE) impedance and resistance results at 31 Hz (±95% confidence intervals).

	Wet F	Wet O	g.tec F	g.tec O	Micro F	Micro O
Impedance	8.2 ± 1.5	19.9 ± 6.0	226.5 ± 62.4	77.9 ± 19.6	36.7 ± 5.7	214.9 ± 33.4
Resistance	8.1 ± 1.4	17.6 ± 4.4	198.2 ± 48.2	70.7 ± 17.5	24.0 ± 2.3	135.8 ± 26.1

**Table 3:** EEG simulation results.

	Correlation (±95% Conf. Int.)		SNR (±95% Conf. Int.)		50 Hz Noise (µV)
	Unfiltered	Filtered	Unfiltered	Filtered	
Generator	0.998 ± 0.005	0.998 ± 0.004	24.93 ± 1.3	25.9 ± 1.3	0.32 ± 0.11
Resistor	0.997 ± 0.015	0.997 ± 0.015	23.4 ± 1.6	23.4 ± 1.6	0.07 ± 0.03
Cloth	0.997 ± 0.015	0.997 ± 0.015	22.7 ± 1.5	23.2 ± 1.6	0.24 ± 0.11
Wet Front.	0.996 ± 0.016	0.997 ± 0.015	21.9 ± 1.5	23.2 ± 1.5	0.36 ± 0.15
Wet Occip.	0.995 ± 0.019	0.997 ± 0.015	20.9 ± 1.6	23.0 ± 1.5	0.51 ± 0.21
g.tec Front.	0.867 ± 0.555	0.982 ± 0.094	6.8 ± 1.5	15.9 ± 1.8	4.94 ± 2.11
g.tec Occip.	0.978 ± 0.107	0.995 ± 0.019	15.1 ± 1.8	21.3 ± 1.6	1.54 ± 0.65
Micro Front.	0.990 ± 0.041	0.996 ± 0.015	18.3 ± 1.7	22.5 ± 1.6	0.94 ± 0.40
Micro Occip.	0.881 ± 0.511	0.985 ± 0.076	7.4 ± 1.5	16.5 ± 1.8	4.59 ± 1.98

**Table 4:** In vivo EEG results.

	g.tec Front.	g.tec Occip.	MicroTIP Front.	MicroTIP Occip
**Correlation**	0.827 ± 0.024	0.855 ± 0.009	0.915 ± 0.014	0.781 ± 0.008
